# Supplementary material for: Fecal Calprotectin in Patients with Crohn’s Disease: A Study Based on the History of Bowel Resection and Location of Disease
Source: Diagnostics (Basel). 2024 Apr 22;14(8):854. doi: 10.3390/diagnostics14080854 (PMC11049016; doi:10.3390/diagnostics14080854)
Supplement: Supplementary file 1 [file diagnostics-14-00854-s001.zip › diagnostics-2951818-Suppl Figure S1.pdf]

**Supplementary Figure S1.**

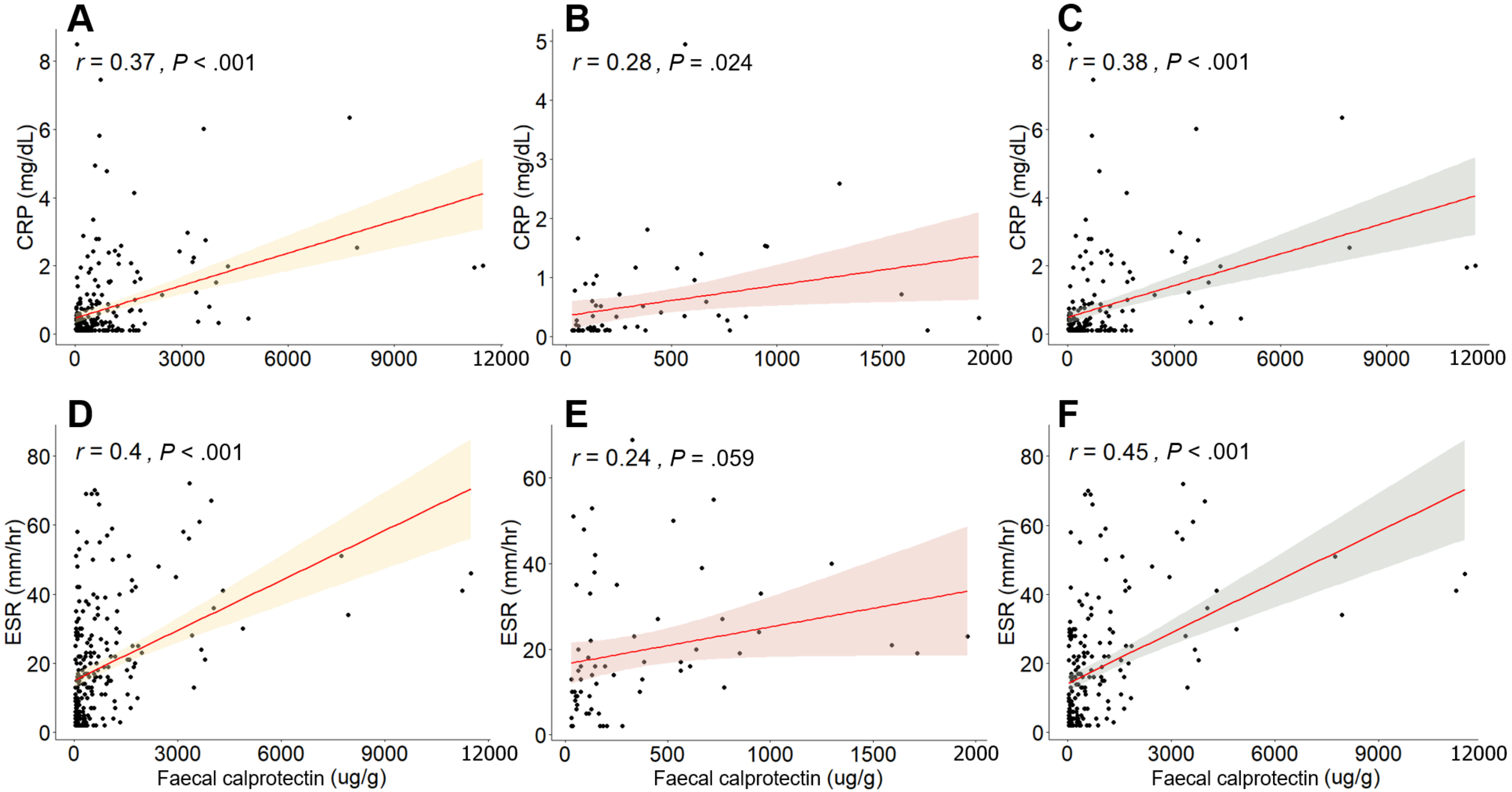

**Supplementary Figure 1.** Scatter plot of (A), (B), (C) CRP against FC, (D), (E), (F) ESR against FC for all, operated patients and non-operated patients, respectively.

Abbreviations: FC, faecal calprotectin; CRP, C-reactive protein; ESR, erythrocyte sedimentation rate
