# Supplementary material for: Fecal Calprotectin in Patients with Crohn’s Disease: A Study Based on the History of Bowel Resection and Location of Disease
Source: Diagnostics (Basel). 2024 Apr 22;14(8):854. doi: 10.3390/diagnostics14080854 (PMC11049016; doi:10.3390/diagnostics14080854)
Supplement: Supplementary file 1 [file diagnostics-14-00854-s001.zip › diagnostics-2951818-Suppl Tables.pdf]

Supplementary Table S1. Diagnostic accuracy of faecal calprotectin, CRP, and CDAI in discriminating between active disease and remission in patients with Crohn' s disease with history of bowel resection

	**Sensitivity (%)**	**Specificity (%)**	**PPV (%)**	**NPV (%)**	**AUROC (95% CI)**
**Patients with history of bowel resection, any location of disease**					
FC cut-off (ug/g)					0.908 (0.860-0.984)
50	89.3	42.9	92.6	33.3	
70.8 (Youden test)	83.9	100.0	100.0	43.8	
100	76.8	100.0	100.0	35.0	
200	46.4	100.0	100.0	18.9	
CRP cut-off (mg/l)					0.735 (0.490-0.893)
0.11 (Youden test)	67.9	85.7	97.4	25.0	
0.25	50.9	85.7	96.6	18.2	
0.5	35.7	85.7	95.2	14.3	
0.8	21.4	85.7	92.3	0.12	
CDAI cut-off					0.620 (0.446-0.783)
66.8 (Youden test)	60.7	71.4	94.4	18.5	
150	33.9	85.7	95.0	14.0	

Patients with history of bowel resection, small bowel involvement only					
FC cut-off (ug/g)					0.867 (0.736-0.962)
50	83.3	42.9	86.2	37.5	
70.8 (Youden test)	76.7	100.0	100.0	0.5	
100	66.7	100.0	100.0	41.2	
200	43.3	100.0	100.0	29.2	
CRP cut-off (mg/l)					0.676 (0.443-0.850)
0.11 (Youden test)	56.7	85.7	94.4	31.6	
0.25	36.7	85.7	91.7	24.0	
0.5	20.0	85.7	85.7	20.0	
0.8	10.0	85.7	75.0	18.2	
CDAI cut-off					0.643 (0.433-0.824)
70.6 (Youden test)	66.7	71.4	90.1	33.3	
150	33.3	85.7	90.1	23.1	

Abbreviations: CRP, C-reactive protein; CDAI, Crohn’s disease activity index; FC, faecal calprotectin; AUROC, area under the receiver operating characteristic curve; CI, confidence interval

Supplementary Table S2. Summary of data from studies on FC in Crohn's disease patients with history of bowel resection[10-12, 28, 29]

Study	Number of patients	Disease evaluation method	FC measurement method	AUROC	Cut-off level (ug/g)	Sensitivity (%)	Specificity (%)
Lobaton, 2013	29	Ileocolonoscopy	Quantum Blue, Bühlmann<br>Calprotectin ELISA, Bühlmann	0.933<br>0.935	272<br>274	79<br>77	97<br>97
Yamamoto, 2013	20	Ileocolonoscopy	Human Calprotectin ELISA Kit, Cell Sciences	-	170	83	93
Lasson, 2014	30	Ileocolonoscopy	Calprotectin ELISA, Bühlmann	-	100	85	35
Boschetti, 2015	86	Ileocolonoscopy	Calprotectin ELISA, Bühlmann	0.86	100	95	54
Wright, 2015	135	Ileocolonoscopy	fCAL ELISA, Bühlmann	0.763	100	89	58
Bachiller, 2016	97	Ileocolonoscopy	Calprest, Eurospital	0.74	60	88	58
Garcia-Planella, 2016	88	Ileocolonoscopy	Calprest, Eurospital	0.75	100	70	64
Lopes, 2016	99	Ileocolonoscopy	EliA Calprotectin, Thermo Fisher Scientific	0.831	100	74	75
Verdejo, 2018	86	Ileocolonoscopy	Quantum Blue, Bühlmann	0.698	62	85.7	45.9
Liu, 2019	90	Enteroscopy	EK-CAL, Bühlmann	0.844	276	96	68
Veyre, 2021	55	Ileocolonoscopy	Calprotectin ELISA, Bühlmann	0.71	65	96	31

Abbreviations: FC, faecal calprotectin; AUROC, area under the receiver operating characteristic curve; ELISA, enzyme-linked immunosorbent assay
