# Supplementary figures and images for: Fecal Calprotectin in Patients with Crohn’s Disease: A Study Based on the History of Bowel Resection and Location of Disease
Source: Diagnostics (Basel). 2024 Apr 22;14(8):854. doi: 10.3390/diagnostics14080854 (PMC11049016; doi:10.3390/diagnostics14080854)

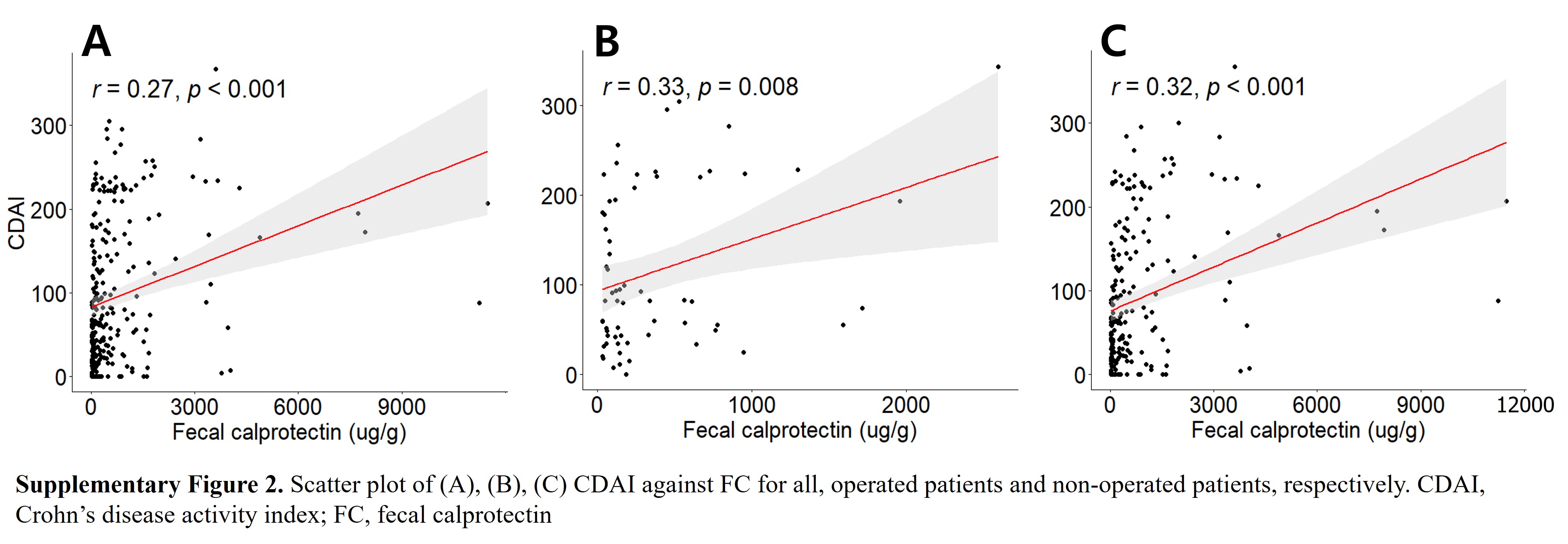

Supplement: Supplementary file 1 [file diagnostics-14-00854-s001.zip › diagnostics-2951818-Suppl Figure S2.jpg]
